# The Ureter’s Midlife Crisis: A Rare Case of Primary Obstructive Megaureter

**DOI:** 10.7759/cureus.82327

**Published:** 2025-04-15

**Authors:** Gowthaam Ramesh, Jatin Soni, Jeyaraman Ramanathan, Arunima Das

**Affiliations:** 1 General Surgery, Sree Balaji Medical College and Hospital, Chennai, IND; 2 Urology, Apollo Hospitals, Chennai, IND; 3 Urology, Sree Balaji Medical College and Hospital, Chennai, IND

**Keywords:** mild hydroureter nephrosis, obstructed megaureter, ureter calculus, ureteric reimplantation, urology department

## Abstract

Congenital megaureter, a rare condition in adults characterized by an abnormally widened ureter, is sometimes diagnosed when individuals develop kidney stones. These stones may form within the dilated ureter itself or in the kidney or ureter above the widened segment. Although uncommon in adults, congenital megaureter should be considered in cases of isolated lower ureteral dilation, as its management may require more complex surgery to prevent recurring symptoms.

## Introduction

The term megaureter, also referred to as hydroureter, large ureter, wide ureter, or megaloureter, describes a ureter that exceeds the normal diameter. This ureteral dilation may occur with or without associated dilatation of the renal pelvis [1]. Megaureter is often associated with congenital abnormalities at the junction where the ureter connects to the bladder [2]. Megaureters are classified into four categories: obstructed, refluxing, refluxing with obstruction, and non-refluxing/non-obstructing. Each category is further subdivided into primary and secondary forms [3]. Adults with this condition typically become symptomatic in their 20s. It more commonly affects a single ureter, usually the left, rather than both ureters [4,5]. While most cases of megaureter do not require surgical intervention, determining whether surgery is needed, and what type, is often complex. Primary obstructive megaureter, a specific subtype, is common in children but rare in adults. It results from a congenital narrowing at the distal ureter where it enters the bladder. Diagnosis typically involves imaging techniques such as X-ray, ultrasound, or MRI, which reveal a dilated ureter with the following characteristics: (a) smooth tapering at the distal end of the ureter, (b) absence of vesicoureteral reflux (backflow of urine toward the kidney), (c) absence of any obstruction beyond the bladder, and (d) cystoscopic confirmation of no visible obstruction at the ureterovesical junction [6]. Spontaneous resolution occurs in more than half of pediatric cases, likely due to maturation and growth of the ureterovesical junction with age [7-9]. In adults, when obstruction causes an obstructive megaureter, it often presents with impaired renal function, nephrolithiasis, or recurrent urinary tract infections (UTIs).

## Case presentation

**Figure 1:**
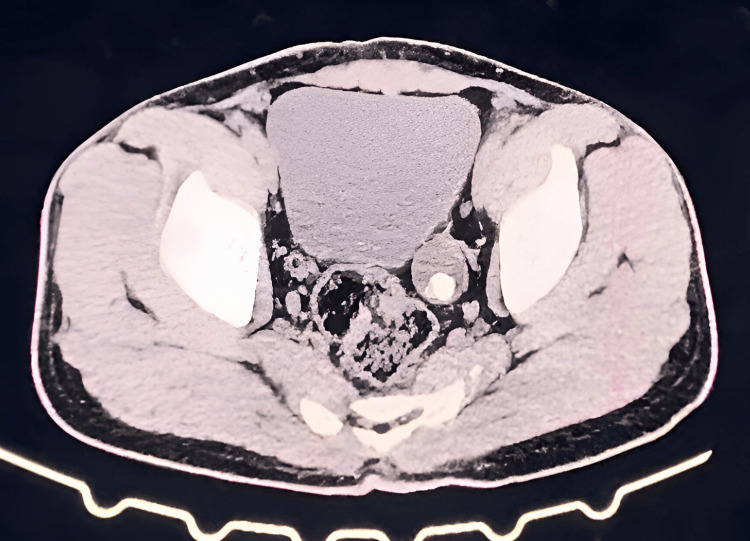
CT urogram showing dilated ureter with calculus

**Figure 2:**
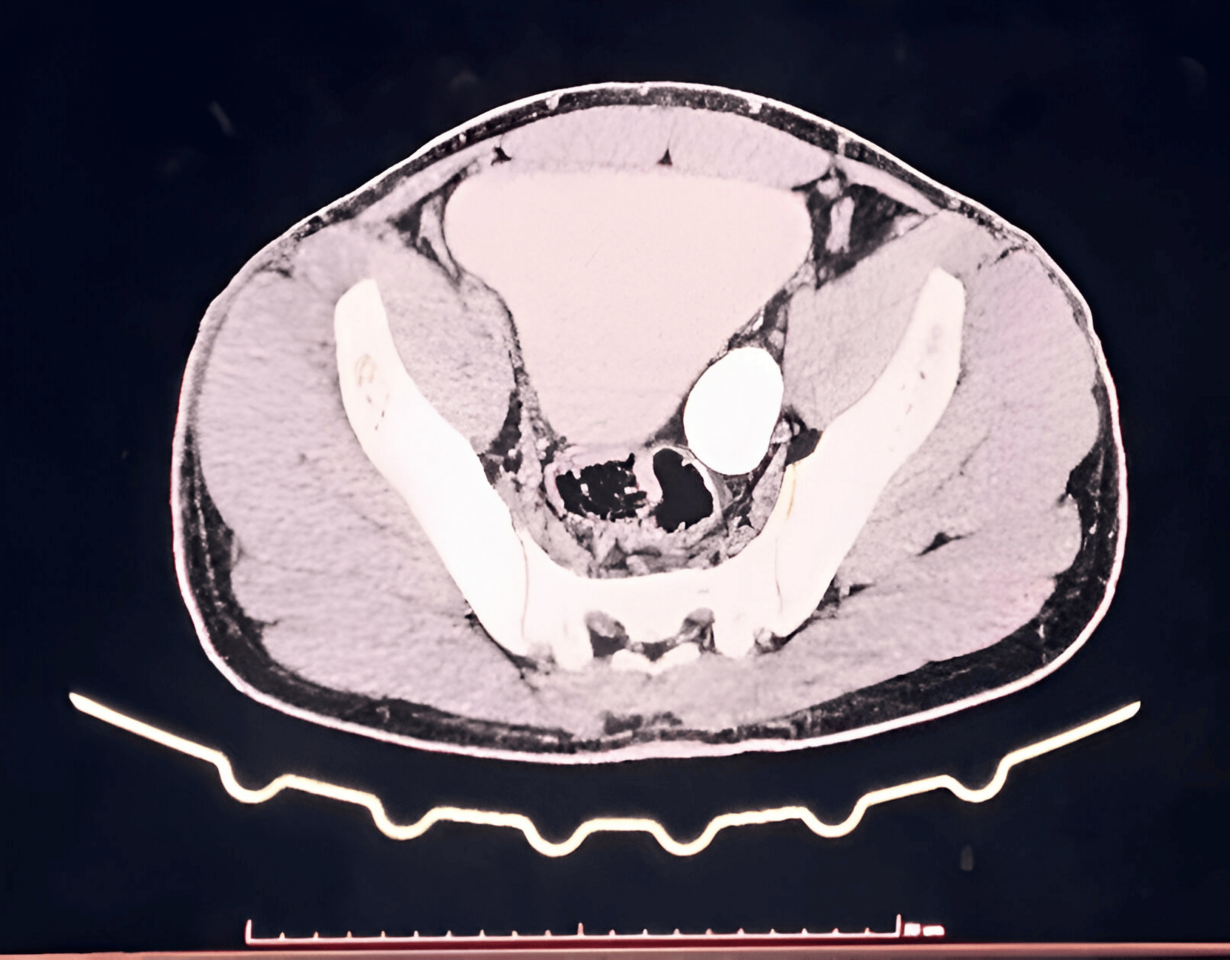
CT urogram showing left megaureter

A 30-year-old male, a chef by occupation, presented with a chief complaint of intermittent left-sided abdominal pain for the past five years. The pain was insidious in onset, colicky in nature, and radiated from the loin to the groin. He had no other specific complaints. On abdominal examination, there was tenderness over the left lumbar region and left iliac fossa, without signs of guarding or rigidity. Renal angle tenderness was also present. Renal function tests were within normal limits. A CT urogram revealed mild left hydroureteronephrosis and moderate-to-gross hydroureter, likely reflux-induced. Additional findings included a left ureteric calculus and a simple renal cyst on the left side, with normal morphology of the proximal ureter (Figure 1 and Figure 2).

**Figure 3:**
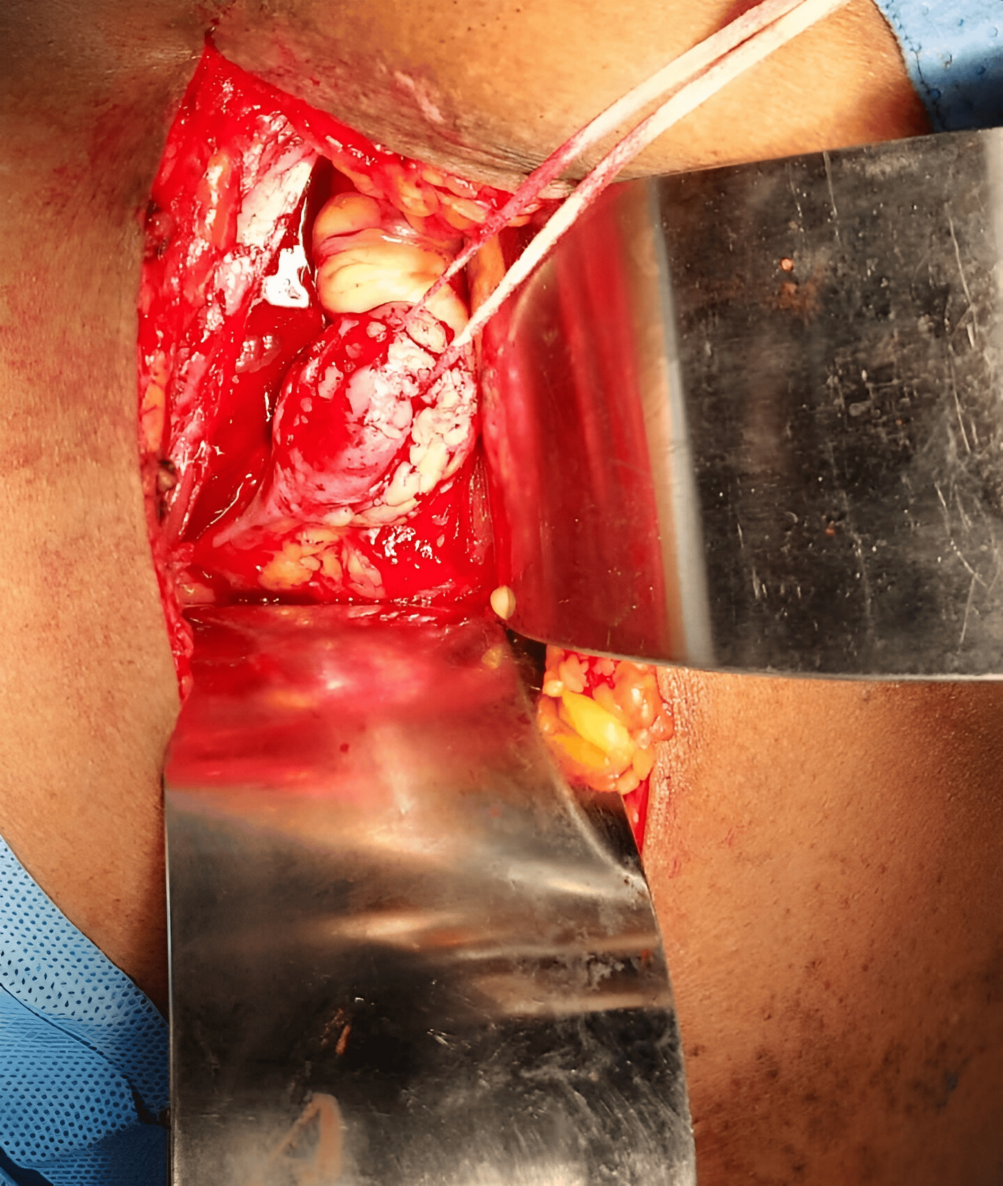
Intraoperative picture of left megaureter A left inguinal Rutherford Morrison muscle-cutting incision was made to access the extraperitoneal space, where the iliac vessels and dilated ureter were visualized near the vessel crossing, medial to the psoas at the level of the sacroiliac joint; the ureter was dissected to the bladder, the obstructed distal segment was excised with double ligation, a proximal ureterotomy was performed to retrieve a single large calculus, followed by tailoring of the left ureter and ureteric reimplantation using the modified Lich-Gregoir technique over a 4.8 Fr, 26 cm DJ stent secured with 3-0 Vicryl.

Left-sided tailoring of the ureter followed by ureteric reimplantation was performed (Figure 3).

**Figure 4:**
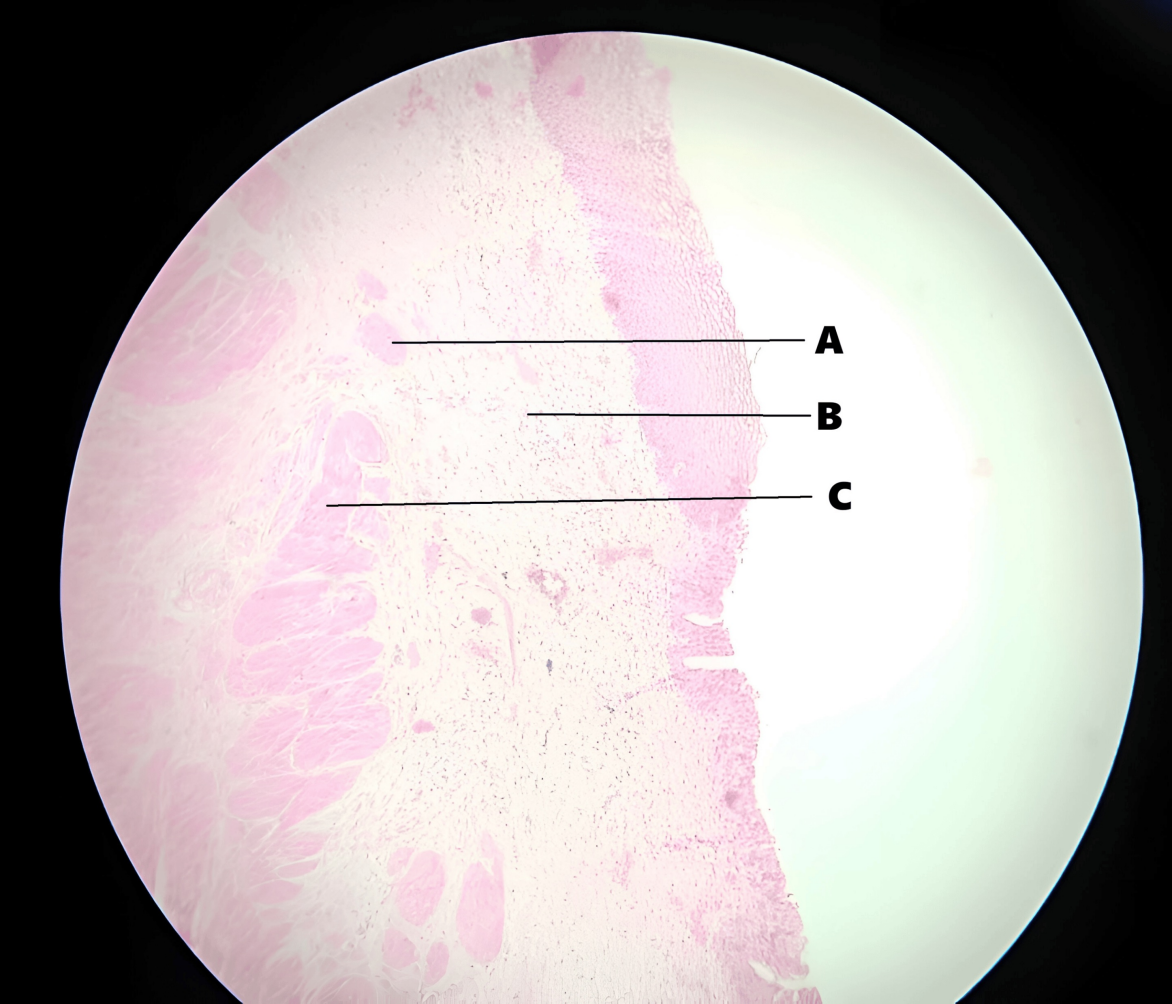
Histopathology slides showing fibrosis, chronic inflammation, and hypertrophy of the muscular layer of the ureteral wall A) Fibrosis of the ureteral wall. B) Inflammatory cells. C) Hypertrophy of the ureteral wall. Magnification, 10x; stain, Hematoxylin and Eosin.

**Figure 5:**
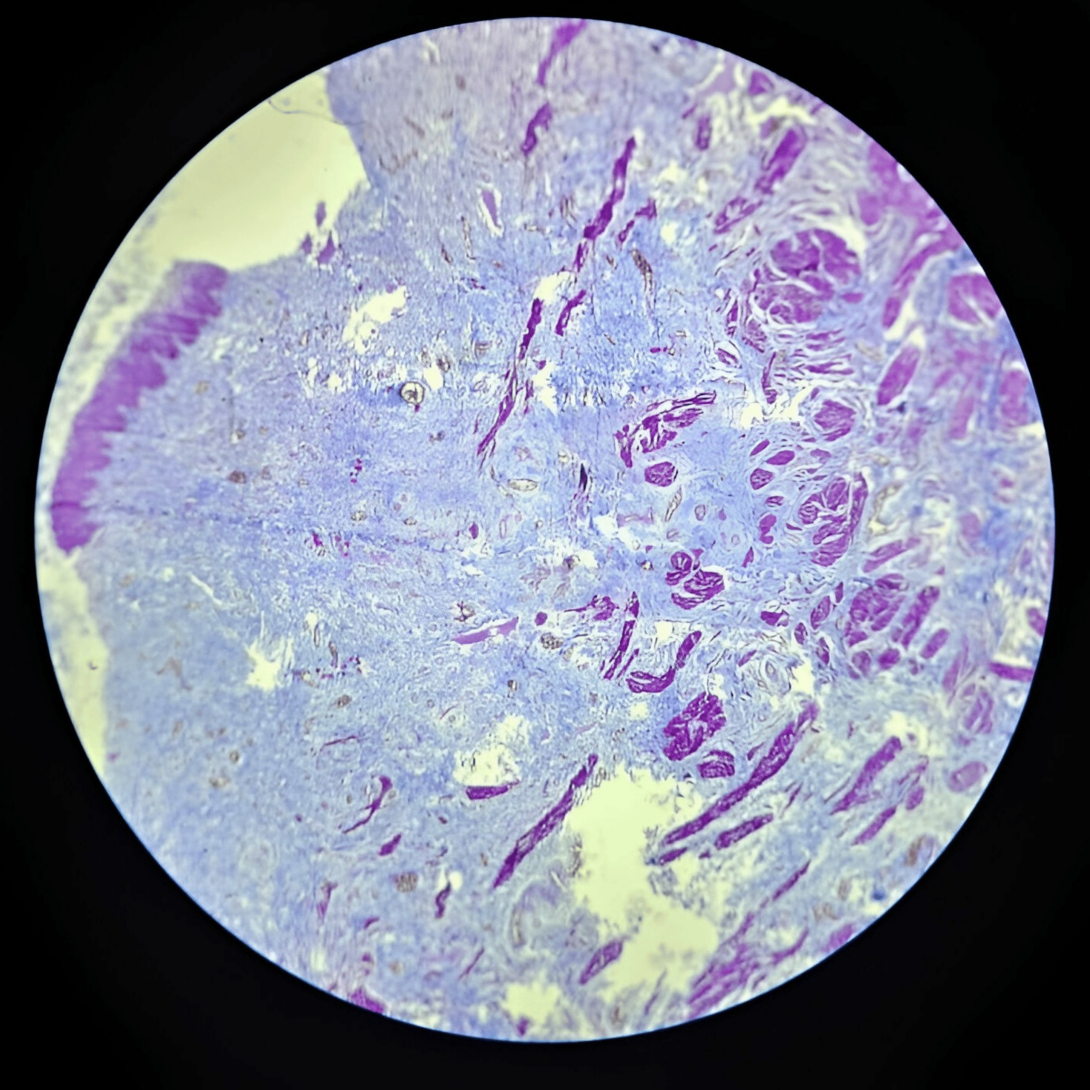
Histopathology of the megaureter Microscopy, 10x; stain, Masson Trichome stain

Histopathology revealed hypertrophy of the muscular layer of the ureteral wall, along with increased fibrosis and chronic inflammation within the ureteral wall (Figure 4 and Figure 5).

On follow-up over one year, no complications were noted.

## Discussion

Primary obstructive megaureter occurs when the lower ureter fails to contract effectively, leading to functional obstruction. This may result from several pathological changes. Excessive collagen deposition can cause scarring and stiffening of the ureteral wall, impairing its ability to propel urine. Weakened musculature, where the ureteral wall muscles are thin or underdeveloped, can lead to ineffective peristalsis. Conversely, muscle hypertrophy, characterized by overly thickened external ureteral muscle layers, may compress the ureter and cause obstruction.

This condition affects approximately 0.36 per 1,000 live births. The clinical presentation of megaureter varies with age: children often present with UTIs and flank pain, while adults more commonly experience hematuria and nephrolithiasis. Notably, nephrolithiasis is a frequent finding in patients with megaureter, with studies reporting its presence in nearly one-third of cases.

Due to potential complications such as nephrolithiasis, recurrent infections, and impaired renal function, various treatment strategies have been proposed. These typically involve resection of the obstructed ureteral segment, followed by ureteroneocystostomy, sometimes accompanied by ureteral remodeling [6].

The etiology of megaureter remains incompletely understood. Some researchers suggest it may stem from abnormalities in the smooth muscle fibers of the ureteral wall or disruptions in collagen synthesis and deposition [10-13].

In this patient, radiographic evaluation revealed marked distal ureteral dilation with relatively normal proximal ureter and renal morphology. This pattern suggests that the observed nephrolithiasis was likely a result of systemic urinary stasis affecting the entire urinary tract, rather than being localized to the dilated ureter. Despite the urinary stasis, renal parenchymal thickness was preserved.

The patient’s mixed uric acid and calcium oxalate nephrolithiasis required surgical intervention. In adult cases of primary obstructive megaureter, key surgical procedures include tailoring of the ureter and ureteric reimplantation. Tailoring involves reducing the caliber of the dilated ureter to restore a more functional diameter and improve urinary flow. Ureteric reimplantation re-establishes the connection between the ureter and the bladder, ensuring a competent, non-obstructive, and reflux-free junction. These procedures aim to relieve symptoms, address complications such as kidney stones and infections, and preserve long-term renal function.

## Conclusions

In adult males, primary obstructive megaureter, though rare, poses significant challenges in both diagnosis and management. Effective treatment depends on accurate diagnosis, typically achieved through a combination of imaging techniques to evaluate the degree of obstruction and its impact on renal function. Surgical intervention remains the primary modality, aiming to relieve the obstruction and preserve kidney function. While outcomes are generally favorable with appropriate management, long-term follow-up is essential to promptly identify and address any potential complications.
